# Genetic evaluation of growth and reproductive traits in Nelore cattle under humid tropical conditions

**DOI:** 10.1007/s11250-026-05278-1

**Published:** 2026-08-12

**Authors:** Elizangela Zayana Lima D´suze, Jhonathan Carvalho Senger, Eula Regina Carrara, Nilsa Duarte da Silva Lima, Jalison Lopes, Henrique Torres Ventura, José Teodoro de Paiva

**Affiliations:** 1 Department of Animal Science, Federal University of Roraima, Boa Vista, Roraima, Brazil; 2 Institute of Animal Science, Sertãozinho, São Paulo, Brazil; 3 Brazilian Association of Zebu Breeders, Uberaba, Minas Gerais Brazil

**Keywords:** Beef cattle, Body weight, Genetic trend, Heritability, Longevity

## Abstract

Assessing the genetic variability of economically important traits is key to guiding selection strategies, and monitoring its changes over time provides critical support for decision-making. The study aimed to estimate genetic parameters and evaluate genetic and phenotypic trends for growth and reproductive traits in Nelore cattle raised under humid tropical conditions. Growth traits included weaning weight (*n* = 1266), yearling weight (*n* = 832), and post-yearling weight (*n* = 733); while reproductive traits included age at first calving (*n* = 639), calving interval (*n* = 1772), and stayability (*n* = 1122), recorded from 2016 to 2022. Variance components were estimated using Bayesian inference, and temporal trends were assessed through linear regression of phenotypic and genetic values on birth year. Growth traits showed moderate to high heritability (ranging from 0.43 to 0.58), indicating good potential for response to selection. Reproductive traits exhibited low heritability (0.04–0.13), reflecting greater environmental influence. Favorable phenotypic trends were observed for weaning weight (3.09 kg/year), yearling weight (2.72 kg/year), post-yearling weight (3.91 kg/year), age at first calving (− 0.68 months/year), and calving interval (− 11.90 days/year). Genetic progress was observed for age at first calving (− 0.03 months/year) and calving interval (− 3.35 days/year), while the remaining traits showed no significant changes. These findings support the use of growth traits for genetic improvement while reinforcing the importance of integrating selection with management strategies to enhance reproductive performance of Nelore cattle under humid tropical conditions.

## Introduction

The cattle herd in the state of Roraima reached 1,056,736 head in 2023, with 99.02% composed of animals genetically suited for beef production (ABIEC 2024). This scenario reflects the consolidation of beef cattle production in the region, where herds are predominantly managed under extensive systems in savanna and transitional forest ecosystems (Braga et al. 2023). In addition to its economic relevance, evidenced by US$ 4.9 million in meat and offal exports in 2024 (SEPLAN 2024), Roraima presents strategic potential for expanding international trade due to its geographic location bordering other countries. Within this production context, the Nelore cattle breed predominates, being widely recognized for its adaptability to tropical environments (Braga et al. 2023).

Despite these favorable conditions, reproductive inefficiency remains one of the primary constraints to improving productivity in tropical beef systems (Claus et al. 2017; Brunes et al. 2020). Reproductive performance in cows directly affects the herd profitability; yet their genetic improvement is inherently challenging due to low heritability and strong environmental influence (Costa et al. 2019; Brzáková et al. 2020). In this context, reproductive traits such as age at first calving, calving interval, and sexual precocity are key factors for enhancing productivity and guiding genetic improvement programs. Females that conceive and calve earlier have a greater potential to produce a higher number of offspring throughout their productive lifespan (Costa et al. 2019). Moreover, females with a lower age at first calving remain less time non-productive within the herd, thereby increasing economic returns for producers (Brunes et al. 2020). Another reproductive trait that has gained prominence in recent years is stayability (Hudson and Van Vleck 1981; Silva et al. 2024), with the most common measure defined as a binary trait in which success corresponds to cows that had three (or more) calving up to 76 months of age (Ramos et al. 2019). Incorporating stayability into genetic evaluation programs enables the selection of sires whose daughters are more likely to exhibit productive longevity, thus improving the efficiency and sustainability of production systems (Ramos et al. 2019; Carvalho et al. 2025).

In contrast, body weight traits, such as weaning, yearling, and post-yearling weights, are key indicators of productive performance in beef cattle systems, as they are directly associated with growth rate and carcass yield. These traits are routinely measured at standardized ages on the farms, particularly in Brazil, where payment systems are largely based on carcass weight (Paterno et al. 2017; Silveira et al. 2024). Thus, economically efficient animals are those that exhibit rapid growth, achieving target weights for weaning, reproduction, or slaughter in fewer days. Because these traits are easy to measure and typically exhibit moderate to high heritability, they allow for faster and more consistent genetic progress through selection (Bessa et al. 2021; Carvalho et al. 2025). Consequently, body weight traits are widely incorporated into selection indices and play a central role in genetic improvement programs for growth performance in Brazilian beef cattle (Bessa et al. 2021; Silveira et al. 2024).

In tropical regions, environmental conditions such as climate variability, pasture quality, and management practices can strongly influence phenotypic expression. Thus, an important challenge in beef cattle production is to balance selection for growth and reproductive performance (Silveira et al. 2024), particularly in humid tropical conditions. Evaluating genetic and phenotypic trends over time allows for the assessment of progress achieved in breeding programs, as well as the animals’ response to management and selection practices adopted over the years (Silveira et al. 2024). This longitudinal approach enables effective monitoring of genetic evolution of the herd and supports future decisions regarding the direction of selection strategies (Carvalho et al. 2025). Such evaluations contribute to characterizing the genetic variability of economically important traits and tracking their evolution over time, providing valuable information for developing more efficient and regionally adapted selection strategies, particularly in challenging environments (Rodrigues et al. 2022).

Thus, the aim of this study was to estimate genetic parameters for weight and reproductive traits, and to evaluate genetic and phenotypic trends over time in Nelore cattle herds raised in extensive systems under the humid tropical conditions in Brazil.

## Materials and methods

### Phenotypic data

The database containing phenotypic and pedigree information of beef cattle was provided by the Brazilian Association of Zebu Breeders (ABCZ). The evaluated animals were distributed across 15 Nelore herds raised in the state of Roraima between 2016 and 2022, and the complete pedigree included 4,721 animals (829 sires and 2,694 dams) over five generations. The dataset included weight traits: weaning weight (WW), yearling weight (YW), and post-yearling weight (PW) for all animals, and reproductive traits: age at first calving (AFC), calving interval (CI), and stayability (STAY) for breeding females. Animal weights were adjusted to standard ages: WW, YW, and PW corresponding to 210, 365, and 450 days of age, respectively. The STAY trait was evaluated at a specific age of 76 months, based on binary observations, where zero (0) indicated failure and one (1) indicated success. A successful phenotype was assigned to cows that had calved at least three times by 76 months of age, while a failure phenotype was assigned to those that did not reach this reproductive threshold.

Data editing was performed through R software (R Core Team 2025), primarily uisng the *dplyr* (Wickham et al. 2024) and *tidyr* packages (Wickham et al. 2023), including exploratory analysis for each trait to identify potential outliers, which were subsequently removed from the dataset. For the execution of genetic analyses, environmental factors (systematic effects) influencing the studied traits were evaluated, and significant effects were identified considering a 5% significance level. The evaluation of systematic effects was conducted by generalized linear models using the R software (R Core Team 2025). For the traits WW, YW and PW the contemporary groups (45, 31, and 30 groups, respectively) were defined by the effects of herd, year, and season of birth. The state of Roraima is characterized by a humid tropical climate with two well-defined seasons: rainy (April to August) and dry (September to March). For the traits AFC, CI, and STAY, the contemporary groups (65, 48 and 83 groups, respectively) were defined by the effects of herd and season of birth of the offspring, as well as year of birth of the offspring for CI. Data consistency was ensured by removing contemporary groups with fewer than three animals.

### Mixed models and heritability estimation

The genetic analysis for WW was conducted by fitting the animal mixed model (Model 1):

Model 1 *y=Xb+Za+Mm+Ppme+e,*

where *y* is the vector of phenotypic observations; *b* is the vector of fixed effects (contemporary groups and dam’s age as a covariate); *a* is the vector of random direct additive genetic effects; *m* is the vector of random maternal additive genetic effects; *pme* is the vector of random permanent maternal environmental effects; *X*, *Z*, *M*, and *P* are the corresponding incidence matrices for the fixed, direct additive genetic, maternal additive genetic, and permanent maternal environmental effects, respectively; and *e* is the vector of random residual effects.

The maternal additive genetic and maternal permanent environmental effects were not considered (Model 2) for YW and PW, as seen in other studies (Bernardes et al. 2015; Carvalho et al. 2025), because the influence of these components is observed until weaning phase (Javier et al. 2024). Likewise, the inclusion of these effects in the model was also not evaluated for the genetic analysis of AFC and STAY (Model Model 2), while Model Model 3 was applied for CI, as described below:

Model 2 *y=Xb+Za+e*

Model 3 *y=Xb+Za+Ppe+e*

where *pe* is the vector of random permanent environmental effects of the animal, and *P* is the incidence matrix relating animals to their permanent environmental effects. For the genetic analysis of STAY, a threshold model was used, in which *y* is the vector of underlying liability on the unobservable scale for the stayability trait.

Variance component estimates were obtained through Bayesian inference using the GIBBSF90 + software (Misztal et al. 2019). A total chain of 500,000 cycles was run, with a burn-in period of 50,000 cycles, and samples were stored every 10 cycles (thinning), resulting in 45,000 posterior samples. Convergence diagnostics of the chain generated by the Gibbs sampler were performed using the Geweke test (Geweke 1991), using the BOA package (Smith 2007) available in the R software (R Core Team 2025).

### Phenotypic and genetic trends

Genetic values for the evaluated traits were predicted using the BLUPF90 + software (Misztal et al. 2019). Phenotypic and genetic trends were estimated through linear regression analyses of the phenotypic and additive genetic values of each animal, respectively, as a function of birth year, according to the following regression equation:

$$\:{y}_{i}={b}_{1}{x}_{1}+\:{b}_{0}+{\epsilon\:}_{i},$$

where *yi* is the predicted genetic value or the phenotypic value of the trait for the *i*th year; *b₁* is the slope (regression coefficient); *xi* is the *i*th birth year; *b₀* is the intercept; and *εi* is the random error.

Regression analyses were performed using the R statistical software (R Core Team 2025), assuming a 5% significance level. For the STAY trait, a generalized linear model with a binomial distribution was used.

## Results and discussion

Statistical parameters for the weight and reproductive traits of Nelore cattle herds raised under humid tropical conditions are shown in Table 1.

**Table 1:** Descriptive statistics for weight and reproductive traits in Nelore cattle herds raised under humid tropical conditions in Brazil

Trait*	Number	Mean	Standard Deviation	Coefficient of variation %	Minimum value	Maximum value	% Success
WW (kg)	1266	196.50	33.64	17.12	120.00	280.00	-
YW (kg)	832	264.70	39.68	14.99	180.00	379.00	-
PW (kg)	733	301.00	41.81	13.89	202.00	399.00	-
AFC (months)	639	36.87	6.04	16.38	22.13	49.73	-
CI (days)	1772	471.00	164.87	35.00	300.00	1607.00	-
STAY	1122	-	-	-	0	1	29.50

*WW = Weaning weight; YW = Yearling weight; PW = Post-yearling weight; AFC = Age at first calving; CI = Calving interval; STAY = Stayability at 76 months

The average weaning weight (WW) was 196.5 ± 33.64 kg, and the yearling weight (YW) was 264.7 ± 39.68 kg, both exceeding the values reported by Rodrigues et al. (2022) for Nelore cattle raised in Northern Brazil (186.20 kg and 235.40 kg, respectively). The average post-yearling weight (PW) was 301.00 ± 41.81 kg, slightly lower than that reported by Arikawa et al. (2025) (310.85 ± 41.01 kg) in cattle from different regions of the country. Regarding reproductive traits, the mean AFC was 36.8 ± 6.04 months, consistent with the value reported by Vitorino et al. (2025) for Mertolenga cattle. The mean CI was 471 ± 164.87 days, shorter than the mean described by Sousa et al. (2015) (591.72 ± 149.91 days). Finally, the STAY trait exhibited a relatively low success rate (29.50%), a value close to that reported by Costa et al. (2019) (26.29%) also in Nelore herds.

## Heritability

The posterior means of variance components, heritabilities, standard deviations, and 95% HPD intervals are shown in Table 2. The convergence diagnostic proposed by Geweke (1991) and the autocorrelation-lag for heritability estimates are reported in Supplementary Table 1. The Geweke diagnostic was not significant (p-value > 0.05) for all parameters, indicating no evidence of lack of convergence in the Markov chain Monte Carlo (MCMC) chains. The relatively wide 95% highest posterior density (HPD) intervals observed for some traits, particularly reproductive traits, reflect the higher uncertainty typically associated with these traits. This is expected due to their low heritability and strong environmental influence. In addition, data structure limitations, including sample size, and pedigree connectivity, may have contributed to the observed dispersion of the posterior estimates. Nevertheless, Bayesian inference is particularly suitable for obtaining robust estimates in situations involving small datasets and complex models (Sorensen and Gianola 2002).

Heritability estimates ranged from 0.04 to 0.58, indicating low to high magnitudes and reflecting varying degrees of genetic and environmental influence on the economic traits evaluated in the Nelore cattle population raised under humid tropical conditions. Although some traits presented moderate to high heritability estimates, Table 2 shows relatively high residual variance for specific traits, suggesting that environmental and other sources of variation had an important effect on phenotypic expression. This pattern is expected in complex traits measured under field conditions, where differences in nutrition, health status, reproductive management, climate, and recording practices may increase environmental heterogeneity. Traits related to body weight exhibited high magnitude, consistent with previous findings that indicate a strong additive genetic contribution to body weight expression in Zebu cattle, ranging from 0.35 to 0.75 (Vaz et al. 2024). The heritability estimate for WW (0.43) was similar that reported by Lopes et al. (2013), who evaluated 33,595 records from Polled Nelore cattle raised on pasture under tropical region. In beef cattle, both direct and maternal additive genetic effects play an important role in traits assessed at young ages, when calves are still dependent on maternal care (Bignardi et al. 2024). Maternal effects refer to differences in preweaning performance resulting from variation in the maternal environment provided by cows during gestation and lactation phases. During the preweaning phase, genetic differences in milk yield may account for approximately half of the variation attributed to maternal effects on calf body weight (Javier et al. 2024).

Besides the great importance of the maternal effect in extensive breeding systems in tropical regions, selection has been focused over the years on heavier weights and on the genetic additive effects (Lopes et al. 2013). Direct heritability estimates for YW (0.58) and PW (0.53) indicate good potential for genetic improvement in Nelore cattle raised under humid tropical conditions. The results obtained in the current study were close to findings by Rodrigues et al. (2022), 0.51 and 0.49, respectively, for YW and PW in Nelore cattle raised in the Northern region of Brazil, and higher than those reported by Carvalho et al. (2025), 0.37 for YW and 0.40 for PW, in Nelore females using the Bayesian approach. Nevertheless, Lopes et al. (2013) cautions that selection for post weaning weights may increase the age at slaughter and the production costs over time. According to these authors, selection of cattle at older ages could result in animals with later growing profiles, whereas early maturing animals would maximize profits from cattle farming. Overall, genetic selection can improve the average performance of Nelore populations under humid tropical conditions, thereby promoting genetic progress in growth traits over time.

In contrast, reproductive traits showed low heritability estimates, indicating a predominance of environmental effects and a slower expected response to selection. The heritability estimate for AFC was low (0.13), suggesting that this trait is strongly influenced by non-genetic factors affecting the onset of reproductive life. Similar estimates have been reported in the literature, including 0.07 for 14,231 Nelore cattle raised in Northeast Brazil (Barbosa et al. 2017) and 0.11 for 18,526 Nelore animals from the Southeast and Mid-west regions of Brazil (Lacerda et al. 2018).Under humid tropical conditions, seasonal variation in forage quality, heat stress, sanitary challenges, and differences in management intensity may increase residual variance and reduce the proportion of phenotypic variance explained by additive genetic effects. The CI trait also showed low heritability (0.04), indicating that environmental variance had a considerable influence on its phenotypic expression. This estimate is consistent with the value of 0.05 reported by Lacerda et al. (2018). Calving interval is highly dependent on postpartum reproductive recovery, body condition score, and nutritional availability. These factors are particularly relevant in pasture-based systems under tropical conditions, where seasonal fluctuations in forage availability may prolong postpartum anestrus and compromise reconception. Therefore, although genetic improvement for shorter calving intervals is possible, the low heritability suggests that progress through selection alone may be slow and that improvements in nutrition, reproductive management, and health control are essential to enhance performance.

The heritability estimated for STAY was 0.13 and aligns with expectations for complex and late-expressing reproductive traits (Kluska et al. 2018; Costa et al. 2020; Ramos et al. 2021). Alternative approaches for evaluating STAY using genomic information in Nelore cattle were investigated by Ramos et al. (2019) and Carvalho et al. (2025), who reported heritabilities estimates ranging from 0.09 to 0.18. The low heritability observed for STAY may be explained by its composite nature, since this trait reflects fertility, longevity, reproductive regularity, health, adaptation, and management decisions related to culling. In addition, because STAY is expressed later in productive life, accumulated environmental effects over successive reproductive cycles may increase residual variance. Overall, reproductive traits were strongly affected by environmental and management-related factors, indicating that genetic progress for reproductive efficiency may be slower and should be supported by improvements in nutrition, reproductive management, health practices, and environmental adaptation. Growth traits showed greater additive genetic influence and, consequently, higher potential for direct genetic gain. In this context, it is important to combine selection strategies with improved management and nutritional practices to enhance growth and reproductive performance in beef cattle raised under humid tropical conditions. Such advances may increase the competitiveness of Nelore cattle production and contribute to global food security (Lopes et al. 2013).

**Table 2:** Posterior means of genetic parameters for weight and reproductive traits in Nelore cattle herds raised under humid tropical conditions in Brazil

Trait*	$\:{\sigma\:}_{g}^{2}$ ± SD [95% HPD]	$\:{\sigma\:}_{m}^{2}$ ± SD [95% HPD]	$\:{\sigma\:}_{pe}^{2}$ ± SD [95% HPD]	$\:{\sigma\:}_{mpe}^{2}$ ± SD [95% HPD]	$\:{\sigma\:}_{e}^{2}$ ± SD [95% HPD]	$\:{\sigma\:}_{p}^{2}$ ± SD [95% HPD]	$\:{h}^{2\:}$± SD [95% HPD]
WW	362.66 ± 81.75 [211.60;524.80]	53.22 ± 31.02 [5.06;114.40]	-	74.21 ± 32.52 [14.14;136.90]	332.030 ± 53.02 [227.60;435.00]	822.12 ± 42.57 [738.97;904.27]	0.43 ± 0.08 [0.27;0.60]
YW	475.92 ± 89.05 [304.50;651.10]	-	-	-	339.44 ± 61.84 [218.40;460.40]	815.36 ± 42.62 [736.00;902.30]	0.58 ± 0.08 [0.41;0.74]
PW	632.27 ± 189.35 [285.60;1016.00]	-	-	-	528.19 ± 138.26 [249.40;792.50]	1160.50 ± 80.33 [1007.30;1319.60]	0.53 ± 0.13 [0.27;0.79]
AFC	3.20 ± 0.06 [0.29;6.24]	-	-	-	20.54 ± 1.71 [17.21;23.90]	23.74 ± 1.39 [21.08;26.50]	0.13 ± 0.06 [0.02;0.25]
CI	1796.20 ± 877.89 [117.70;3382.00]	-	768.44 ± 607.56 [261.20;1964.00]	-	20672.00 ± 941.50 [18870.00;22570.00]	23237.00 ± 842.62 [21607.00;24886.00]	0.04 ± 0.03 [0.01;0.08]
STAY	0.14 ± 0.11 [0.01;0.32]	-	-	-	0.89 ± 0.10 [0.70;1.09]	1.02 ± 0.16 [0.75;1.35]	0.13 ± 0.01 [0.01;0.26]

*SD = standard deviation; 95% HPD = 95% highest posterior density interval; $\:{\sigma\:}_{g}^{2}$ = direct additive genetic variance; $\:{\sigma\:}_{m}^{2}$ = maternal additive genetic variance; $\:{\sigma\:}_{pe}^{2}$ = permanent environmental variance; $\:{\sigma\:}_{mpe}^{2}$ = maternal permanent environmental variance; $\:{\sigma\:}_{e}^{2}$ = residual variance; $\:{\sigma\:}_{p}^{2}$ = phenotypic variance; $\:{h}^{2\:}$= heritability; WW = weaning weight; YW = yearling weight; PW = Post-yearling weight; AFC = age at first calving; CI = calving interval; STAY = stayability at 76 months

### Phenotypic and genetic trend

Phenotypic trends were positive for all evaluated traits (p-value < 0.05, Table 3), indicating that the improvements observed in Nelore herds under humid tropical environment reflect both enhanced environmental and management conditions, in addition to genetic potential achieved in the population.

**Table 3:** Linear regression coefficients for weight and reproductive traits over the year of birth in Nelore cattle herds raised under humid tropical conditions in Brazil

Trait*	Phenotypic Trend		Genetic Trend	
	b_0_ (*p*-value)	b_1_ (*p*-value)	b_0_ (*p*-value)	b_1_ (*p*-value)
WW (kg)	-6049.74 (< 0.001)	3.092 (< 0.001)*	-819.77 (0.167)	0.408 (0.165)^ns^
YW (kg)	-5238.93 (0.19)	2.724 (0.014)*	-537.45 (0.480)	0.267 (0.477) ^ns^
PW (kg)	-761.27 (0.012)	3.916 (0.009)*	1150.74 (0.276)	-0.568 (0.277) ^ns^
AFC (months)	1413.40 (< 0.001)	-0.683 (< 0.001)*	73.28 (0.019)	-0.036 (0.019)*
CI (days)	24457.27 (< 0.001)	-11.905 (< 0.001)*	6748.97 (< 0.001)	-3.352 (< 0.001)*
STAY	1317.91 (< 0.001)	-0.654 (< 0.001)*	-8.48 (0.306)	0.004 (0.307) ^ns^

*WW = Weaning weight; YW = Yearling weight; PW = Post-yearling weight; AFC = Age at first calving; CI = Calving interval; STAY = Stayability at 76 months; * = p-value < 0.05; ns = p-value > 0.05

**Fig. 1:**
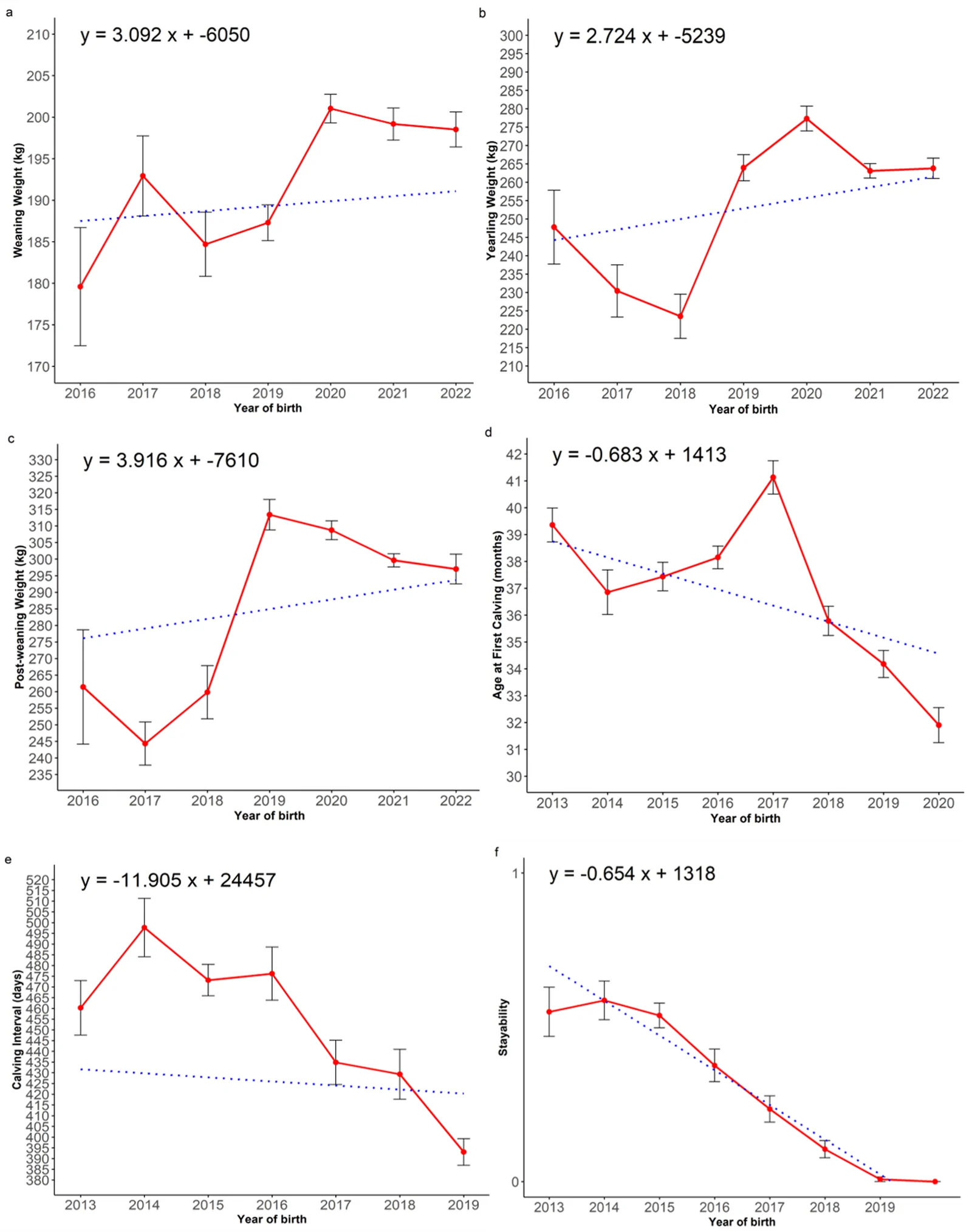
Phenotypic trend (linear regression/dotted line) for weaning weight (**a**), yearling weight (**b**), post-yearling weight (**c**), age at first calving (**d**), calving interval (**e**) and stayability (logistic regression) (**f**), with their respective means and standard deviations of phenotypic values over the years (solid line) (p-value < 0.05)

The phenotypic trend for WW showed a slight increase over the evaluated period, with an average annual gain of 3.091 kg (Fig. 1a). Although fluctuations between years were minimal, overall stability may be associated with consistent management practices during the early stages of calf development in the region. Moreover, this pattern also reflects gradual improvements in the dams’ maternal ability. For YW, the phenotypic trend was also positive, with an average annual increase of 2.724 kg (Fig. 1b). Although some fluctuations were observed, particularly a decline in 2018 followed by recovery in subsequent years, this pattern likely reflects variations in nutritional management during this growth phase, as well as a degree selection pressure directed toward traits related to body weight.

The highest rate of increase was observed for PW, whose phenotypic annual trend was 3.916 kg (Fig. 1c). This result demonstrates the most expressive phenotypic progress for this stage of animal development, likely reflecting both the intensity of selection applied and improvements in feeding and supplementation management during the final growth phase. Similar findings have been reported in other studies, with genetic gains for post-yearling weight (1.90 kg/year) in Nelore cattle (Barbosa et al. 2017). Conversely, Lopes et al. (2009) reported lower phenotypic progress for WW and PW in Brangus cattle from 1987 to 2001. Likewise, Rodrigues et al. (2022) reported substantial phenotypic progress for WW and YW in Nelore cattle raised in the Northern region of Brazil. According to these authors, selection for post-weaning weights in the Northern region has been prioritized, which over the years may lead to higher production costs, increased slaughter age, and challenges in achieving adequate carcass finishing. Therefore, it is recommended that greater emphasis be placed on selecting animals with superior pre-weaning performance, as this phase corresponds to the period of highest growth rate and physiological development.

Age at first calving (AFC) showed a negative phenotypic trend, with an average reduction of − 0.68 months per year (Fig. 1d), which is a desirable outcome in production systems aimed at sexual precocity and greater reproductive efficiency. The reduction in AFC observed in Nelore herds raised under humid tropical conditions can be partially attributed to improvements in management practices adopted at the farm. These advances include the use of genetically superior sires, which contributed to enhanced reproductive precocity, as well as strategic nutritional supplementation, especially during critical growth phases, supporting adequate body development, and enabling heifers to reach puberty earlier. Adequate energy intake and balanced levels of amino acids, vitamins, and minerals are essential for the initiation of the estrous cycle and reproductive activity (Marrella et al. 2021). Additionally, improvements in herd management, particularly in health protocols, have likely played an important role in accelerating heifer development and increasing reproductive efficiency. Similarly, negative trends have been reported in other Nelore herds raised in the Midwestern, Northern, and Northeastern regions of Brazil (Magnabosco et al. 2016; Silva et al. 2018). The phenotypic trend showed a decline in calving interval values over the evaluated years, with an average reduction of 11.9 days per year (Fig. 1e), reflecting improved efficiency in the resumption of postpartum reproductive activity of the dams. Similarly, Bene et al. (2021, 2022) reported a slight decrease in calving interval in Limousin cattle between 1996 and 2016, supporting the pattern observed in the present study.

Conversely, the STAY trait exhibited a more pronounced negative phenotypic trend (Fig. 1f), indicating a reduction in the probability of productive longevity among females in the herd over the years. This pattern may be associated with management or challenges related to health, the increased replacement of cows by younger heifers, or lack of selection pressure for reproductive longevity in the Nelore herds evaluated humid tropical conditions. Carvalho et al. (2025) reported positive genetic trends in Nelore females while Bessa et al. (2021) found negative annual average trend. These findings highlight the need for more comprehensive studies which accounts for additional environmental variables, particularly those related to management practices, which variables potentially affecting the retention of breeding females in production system. Although the reduction in age at first calving reflects improved reproductive precocity, it may be accompanied by additional challenges in Nelore herd management. Nutritional management is one of the main factors affecting reproductive performance in beef cattle. In Brazilian production systems, calving is often concentrated during the dry season and the transition to the rainy season, when forage quality is typically low (Catussi et al. 2022). Consequently, beef cows frequently experience inadequate nutrient intake during the peripartum period. Combined with the increased energy and protein demands of late gestation, this can lead to low body condition score at calving and negative energy balance in early postpartum, impairing reproductive performance by extending the postpartum anestrous interval and reducing pregnancy rates (Catussi et al. 2022). These effects are more pronounced in primiparous cows, which face additional nutritional demands for growth, lactation, and subsequent pregnancy. After first calving, these animals are particularly challenged under humid tropical conditions, where forage availability and quality fluctuate seasonally. Inadequate nutritional management during this period may compromise body condition score maintenance, delay postpartum recovery, and reduce reconception rates (Boligon et al. 2012; Valente et al. 2017).

**Fig. 2:**
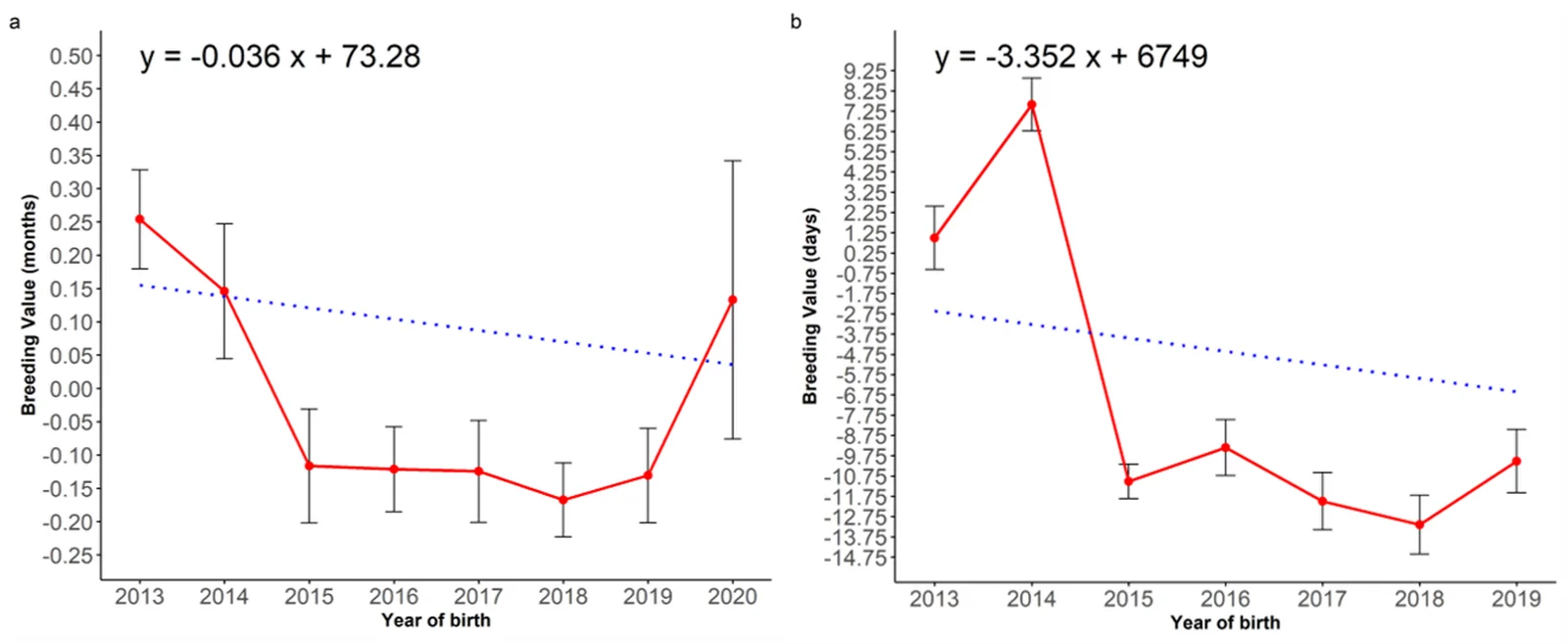
Genetic trend (linear regression/dotted line) for age at first calving (**a**) and calving interval (**b**), with their respective means and standard deviations of genetic values over the years (solid line) (p-value < 0.05)

Genetic trends were significant (p-value < 0.05) for both AFC and CI (Table 3), with regression coefficients of − 0.036 months (Fig. 2a) and − 3.35 days (Fig. 2b), respectively.

Thus, these results demonstrate that Nelore cattle herds raised under humid tropical conditions have evolved over the years, showing favorable genetic progress for these two reproductive traits evaluated. Similarly, Magnabosco et al. (2016) reported decreasing genetic values for these reproductive traits, whereas other studies reported a slight increase in calving interval (Nepomuceno et al. 2012; Barbosa et al. 2017). In contrast, genetic trends for the body weight traits and for stayability were not significant (p-value > 0.05) (Table 3). The absence of significant annual genetic trends for the remaining traits may be attributed to the limited data availability or the strong influence of environmental factors. In this context, greater attention should be directed toward the selection criteria applied in Nelore herds, particularly emphasizing cow longevity and higher productivity, traits that are directly related to sustainability and overall efficiency of production systems.

This study evaluated genetic parameters and trends for growth and reproductive traits in Nelore cattle under humid tropical conditions, where genetic variability and management challenges can strongly influence animal performance. Weight traits exhibited moderate to high heritability, indicating potential for genetic improvement, whereas reproductive traits were more influenced by environmental factors. Favorable phenotypic trends were observed for both growth and reproductive traits, along with genetic progress for age at first calving and calving interval. These findings are particularly relevant for tropical systems, where improving reproductive efficiency remains a major constraint to productivity. Future studies should prioritize larger datasets and investigate genotype-environment interactions to enhance the efficiency and sustainability of tropical beef production systems.

## Data Availability

The datasets analyzed in this study are not publicly available but can be obtained from the corresponding author upon reasonable request.
